# *Post‐hoc* safety/efficacy analyses from pediatric delgocitinib atopic dermatitis trials

**DOI:** 10.1111/ped.15798

**Published:** 2024-10-07

**Authors:** Tatsuki Fukuie, Hiroyuki Toyama, Mai Tanaka, Katsuyo Ohashi‐Doi, Kenji Kabashima

**Affiliations:** ^1^ Allergy Center, National Center for Child Health and Development Tokyo Japan; ^2^ Medical Affairs Department TORII Pharmaceutical Co Ltd Tokyo Japan; ^3^ Department of Dermatology, Graduate School of Medicine Kyoto University Kyoto Japan

**Keywords:** atopic dermatitis, delgocitinib, JAK inhibitor, pediatric atopic dermatitis, safety and efficacy

## Abstract

**Background:**

Delgocitinib ointment is usually recommended for use in children at a concentration of 0.25%. However, there are no clear criteria for dosing, except that a 0.5% formulation may also be used, depending on symptom severity. Treatment of atopic dermatitis is based on combinations of topical corticosteroids, tacrolimus ointment, and delgocitinib ointment, but there are no reports on the safety of delgocitinib ointment when used in combination with other drugs.

**Methods:**

This is a *post‐hoc* analysis of data from two delgocitinib ointment trials with pediatric atopic dermatitis patients. The efficacy and safety of the 0.25% and 0.5% formulations were compared. Efficacy and safety were evaluated after up to 4 and 56 weeks of treatment, respectively. The safety of delgocitinib ointment when used in combination with topical corticosteroids and/or tacrolimus ointment was investigated.

**Results:**

The dose–response relationship was examined according to baseline disease severity. The proportions of subjects with mild disease who achieved cumulative investigator's global assessment of 0 (clear) or 1 (almost clear) were 46.2% (0.25% ointment), 71.4% (0.5% ointment), and 7.7% (vehicle). For subjects with moderate to severe disease, the corresponding proportions were 19.0%, 20.0%, and 0.0%, respectively. No overall differences were seen in the safety profiles of the 0.25% and 0.5% delgocitinib ointment doses, or in the safety profiles of the two doses relating to disease severity or to concomitant use of topical corticosteroids and/or tacrolimus ointment.

**Conclusions:**

These analyses indicate that after up to 4 weeks of treatment, delgocitinib 0.5% ointment may be more effective than the 0.25% dose for mild atopic dermatitis, and that after up to 56 weeks of treatment, delgocitinib is well tolerated in a pediatric trial population when used as prescribed in combination with topical corticosteroids and/or tacrolimus ointment.

## INTRODUCTION

The purposes of this article were to compare the efficacy and safety of two doses (0.25% and 0.5%) of delgocitinib in children with atopic dermatitis (AD) and to investigate the safety of delgocitinib ointment when used in combination with topical corticosteroids or tacrolimus ointment. *Post‐hoc* analyses of data from two double‐blind placebo‐controlled delgocitinib ointment trials with pediatric AD patients were conducted.

AD is a common chronic inflammatory dermatological disease that affects 15 to 30% of children and 2 to 10% of adults in industrialized countries.^1^ It typically affects individuals with a family history of atopy^2^ and is characterized by three main manifestations: intense pruritus, eczematous skin lesions, and dry skin caused by skin barrier disruption.^3^ A characteristic of AD is the fluctuation of symptoms between remissions and relapses. These fluctuations demand particular attention to treatment regimens and appropriateness of medications. The most important factor in the treatment of inflammation in AD is to calm the inflammation quickly and reliably, and the selection and combination of topical corticosteroids (TCS), tacrolimus ointment, delgocitinib ointment and difamilast ointment are fundamental to this treatment.

Delgocitinib is available in Japan as an ointment formulation in two concentrations, 0.25% and 0.5%, and is indicated for mild to severe AD in adults and children (6 months and older).^4^, ^5^ According to the delgocitinib package insert,^5^ the 0.25% ointment is the normal dose for pediatric use but it also states that the 0.5% ointment may be used for children when symptoms are severe or if the response to the 0.25% ointment is inadequate.^5^

When using delgocitinib ointment in clinical practice, the two most important concerns are the difference in safety and efficacy of the two available doses and the safety of using delgocitinib ointment concomitantly with other medications. As the current delgocitinib ointment treatment guidelines were based on the results from clinical trials with AD patients 16 years or older,^6^ analyses of safety and efficacy in pediatric populations would provide useful information for the prescribing physicians. Two clinical trials have been conducted recently to examine the safety and efficacy of delgocitinib ointment in pediatric populations—trial QBB2‐1 (JapicCTI‐173553)^7^ and trial QBB4‐1 (JapicCTI‐184064)^8^—but these trials did not include detailed examination of safety and efficacy in relation to delgocitinib dose or to the use of concomitant medication.

This report provides *post‐hoc* analyses of data from the QBB2‐1 and QBB4‐1 trials to examine the efficacy of delgocitinib 0.25% and 0.5% ointment according to disease severity at baseline. The frequency of occurrence of treatment‐related adverse events (TRAEs) that coincided with the use of 0.25% and 0.5% delgocitinib ointment, respectively, was analyzed. Finally, the frequency of TRAEs in patients who received rescue medication (topical corticosteroids and/or tacrolimus ointment) concomitantly with delgocitinib ointment treatment was compared with the frequency of TRAEs in patients receiving only delgocitinib treatment.

## METHODS

### Study designs

**FIGURE 1:**
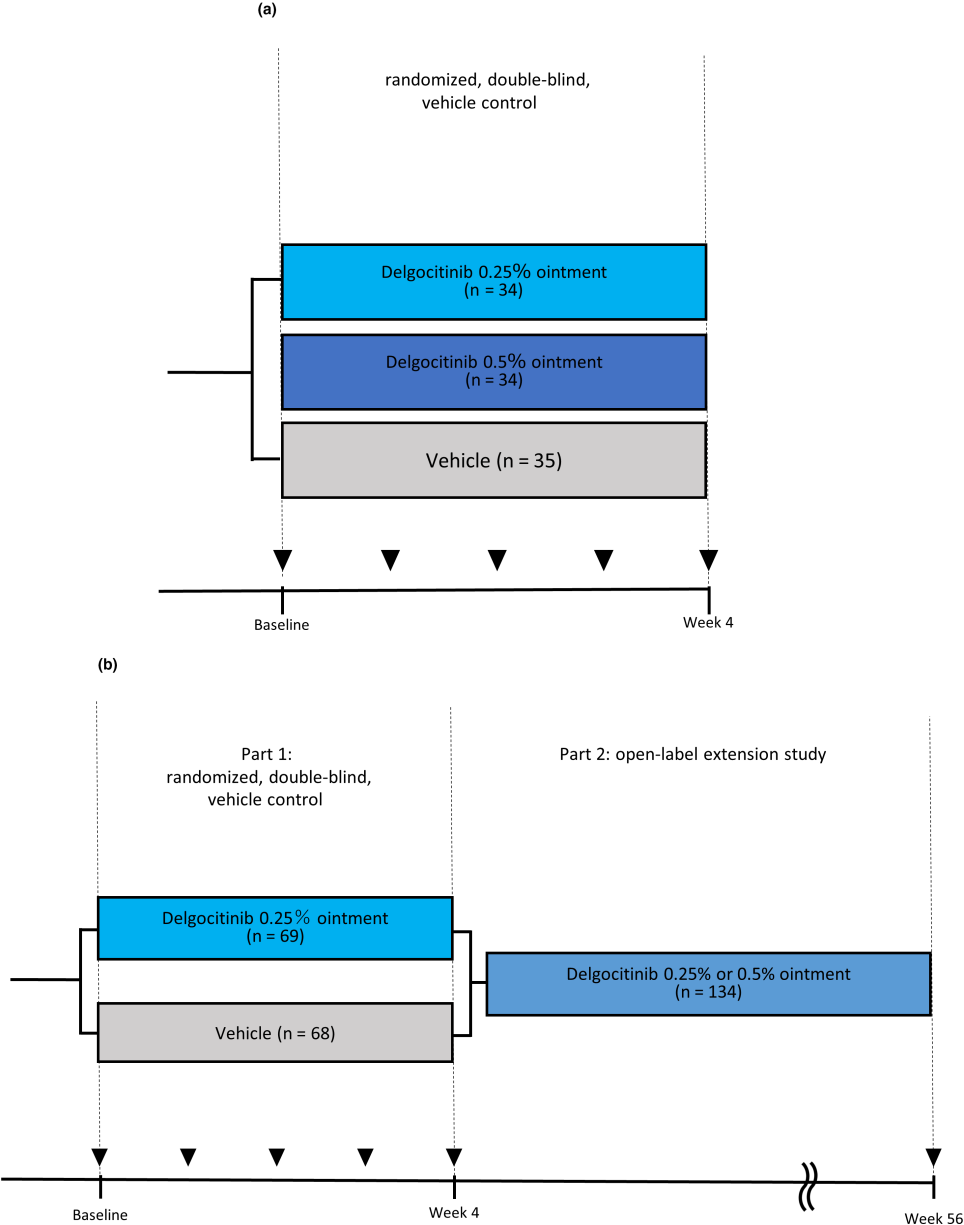
(a) QBB2‐1 and (b) QBB4‐1 trial designs.

This *post‐hoc* analysis used the results of two reported clinical trials in pediatric AD patients (2–15 years old):

1. QBB2‐1 (JapicCTI‐173553, *n* = 103, age: 2–15 years)^7^ was used to examine differences in efficacy and safety between 0.25% and 0.5% formulations in pediatric AD patients. The study design is shown in Figure 1a.
2. QBB4‐1 (JapicCTI‐184064, *n* = 137, age: 2–15 years)^8^ was used to examine the safety of this ointment in combination with other drugs.

The study design is shown in Figure 1b. The details of QBB2‐1 and QBB4‐1 are provided in the respective papers.

### Cumulative investigator's global assessment (IGA) 0,1 (QBB2‐1)

AD is a chronic disease that repeatedly relapses and remits so it was considered important to judge efficacy over the entire study period rather than at a single point in time. In this *post‐hoc* analysis, efficacy was therefore evaluated as the cumulative IGA 0,1 achievement rate.

Patients who received delgocitinib ointment or vehicle, and who had at least one efficacy evaluation, were included in the efficacy analysis population. Cumulative IGA 0,1 was assessed as the percentage of patients who achieved IGA 0 or 1 at least once during the 4‐week treatment period. Cumulative IGA 0,1 was evaluated by treatment group (0.25% delgocitinib ointment, 0.5% delgocitinib ointment, and vehicle) and by IGA status (IGA 2 or IGA 3,4) at baseline.

### Safety assessments (QBB2‐1 and QBB4‐1)

In both trials, patients with at least one safety investigation who had received study medication were included in the safety analysis population. In QBB2‐1, treatment‐related adverse events (TRAEs) by system organ class (SOC) and preferred term (PT) (MedDRA/J version 19.1 classification scheme) were evaluated by treatment group (0.25% delgocitinib ointment, 0.5% delgocitinib ointment, and vehicle). In QBB4‐1, TRAEs by SOC and PT (MedDRA/J version 21.0) were evaluated by the delgocitinib dose used at the time of onset of a TRAE (as also included in Nakagawa et al.^8^), and by rescue medication use.

## RESULTS

### QBB2‐1 study population

Overall, the QBB2‐1 study population was well‐balanced regarding sex, age, duration of AD prior to the study, disease severity, the use of pretreatment drugs, and the number of patients in each treatment arm (0.25% delgocitinib ointment, 0.5% delgocitinib ointment, and vehicle) (Table 1). Two subgroups of the QBB2‐1 study population were defined according to disease severity with either IGA 2 (mild disease) or IGA 3,4 (moderate–severe disease) at baseline (Table 1). Proportionally more patients were identified with IGA 3,4 than IGA 2 at baseline, but the two subgroups were similar regarding age and duration of AD prior to the study (Table 1). As expected, modified Eczema Area and Severity Index (mEASI) scores and use of pretreatment drugs were higher in the IGA 3,4 group than the IGA 2 group (Table 1).

**TABLE 1:** QBB2‐1 demographics and baseline characteristics by IGA score.

Characteristics	IGA 2 (mild)			IGA 3,4 (moderate and severe)			Total		
	Vehicle	Delgocitinib 0.25% ointment	Delgocitinib 0.5% ointment	Vehicle	Delgocitinib 0.25% ointment	Delgocitinib 0.5% ointment	Vehicle	Delgocitinib 0.25% ointment	Delgocitinib 0.5% ointment
*n*	13	13	14	22	21	20	35	34	34
Sex (*n* [%])									
Men	4 (11.4)	10 (29.4)	6 (17.6)	14 (40.0)	12 (35.3)	12 (35.3)	18 (51.4)	22 (64.7)	18 (52.9)
Women	9 (25.7)	3 (8.8)	8 (23.5)	8 (22.9)	9 (26.5)	8 (23.5)	17 (48.6)	12 (35.3)	16 (47.1)
Age (years, mean ± SD)	9.2 ± 4.3	8.7 ± 3.6	6.7 ± 4.9	8.3 ± 3.9	8.3 ± 4.0	9.8 ± 3.7	8.6 ± 4.0	8.4 ± 3.8	8.5 ± 4.4
Duration of AD (years, mean ± SD)	6.3 ± 4.6	5.9 ± 4.6	5.6 ± 4.6	6.5 ± 4.1	6.2 ± 3.8	7.4 ± 3.2	6.4 ± 4.2	6.1 ± 4.0	6.6 ± 3.8
mEASI score BL (mean ± SD)	8.6 ± 2.9	8.6 ± 2.9	8.3 ± 2.4	12.8 ± 4.9	11.6 ± 4.5	13.1 ± 4.8	11.3 ± 4.7	10.5 ± 4.2	11.1 ± 4.6
Pretreatment drug (*n* [%])	10 (76.9)	10 (76.9)	9 (64.3)	20 (90.9)	20 (95.2)	19 (95.0)	30 (85.7)	30 (88.2)	28 (82.4)
Topical corticosteroids (*n* [%])	9 (69.2)	10 (76.9)	8 (57.1)	20 (90.9)	20 (95.2)	18 (90.0)	29 (82.9)	30 (88.2)	26 (76.5)
Strongest	0 (0.0)	0 (0.0)	0 (0.0)	0 (0.0)	0 (0.0)	0 (0.0)	0 (0.0)	0 (0.0)	0 (0.0)
Very strong	2 (15.4)	0 (0.0)	0 (0.0)	5 (22.7)	3 (14.3)	4 (20.0)	7 (20.0)	3 (8.8)	4 (11.8)
Strong	7 (53.8)	6 (46.2)	4 (28.6)	10 (45.5)	13 (61.9)	7 (33.3)	17 (48.6)	19 (55.9)	11 (32.4)
Medium	7 (53.8)	7 (53.8)	7 (50.0)	15 (68.2)	13 (61.9)	14 (70.0)	22 (62.9)	20 (58.8)	21 (61.8)
Weak	0 (0.0)	0 (0.0)	0 (0.0)	0 (0.0)	0 (0.0)	1 (5.0)	0 (0.0)	0 (0.0)	1 (2.9)
Tacrolimus ointment (*n* [%])	2 (15.4)	0 (0.0)	1 (7.1)	6 (27.3)	5 (23.8)	7 (35.0)	8 (22.9)	5 (14.7)	8 (23.5)

Abbreviations: AD, atopic dermatitis; BL, baseline; IGA, investigator’s global assessment; mEASI, modified eczema area and severity index; SD, standard deviation.

### QBB4‐1 study population

The overall QBB4‐1 patient demographics and baseline characteristics are described in Nakagawa et al.^8^ Here, two subgroups of the QBB4‐1 study population were defined according to rescue medication use (Table 2). During Part 2 of the study, 62 (46.3%) patients completed the trial without any use of rescue medication and the allowed rescue medications (tacrolimus ointment and/or TCS) were used by 72 patients (53.7%) (Table 2). The two subgroups were well balanced regarding sex, age, duration of AD prior to the study, mEASI score at baseline, and number of patients in each group (Table 2). There were more patients with IGA 3 (moderate disease) and IGA 4 (severe disease) at baseline in the subgroup of patients that used rescue medication during Part 2 of the study than in the subgroup of patients who did not receive any rescue medication (Table 2). For patients with IGA 2 (mild disease) at baseline, there were proportionally more patients in the no‐rescue medication subgroup (Table 2).

**TABLE 2:** QBB4‐1 demographics and baseline characteristics by rescue medication use.

Characteristics	Without rescue medication	With rescue medication	Total
*n* (%)	62 (46.3)	72 (53.7)	134 (100.0)
Sex (*n* [%])			
Men	30 (48.4)	38 (52.8)	68 (50.7)
Women	32 (51.6)	34 (47.2)	66 (49.3)
Age (years, mean ± SD)	8.5 ± 4.0	8.1 ± 3.6	8.3 ± 3.8
Duration of AD (years, mean ± SD)	5.7 ± 3.7	6.2 ± 3.7	6.0 ± 3.7
mEASI score BL (mean ± SD)	9.5 ± 5.1	11.9 ± 5.5	10.8 ± 5.4
IGA score BL (*n* [%])			
2 (mild)	23 (37.1)	7 (9.7)	30 (22.4)
3 (moderate)	28 (45.2)	43 (59.7)	71 (53.0)
4 (severe)	11 (17.7)	22 (30.6)	33 (24.6)

Abbreviations: AD, atopic dermatitis; BL, baseline; IGA, investigator’s global assessment; mEASI, modified eczema area and severity index; SD, standard deviation.

### Efficacy assessment

**FIGURE 2:**
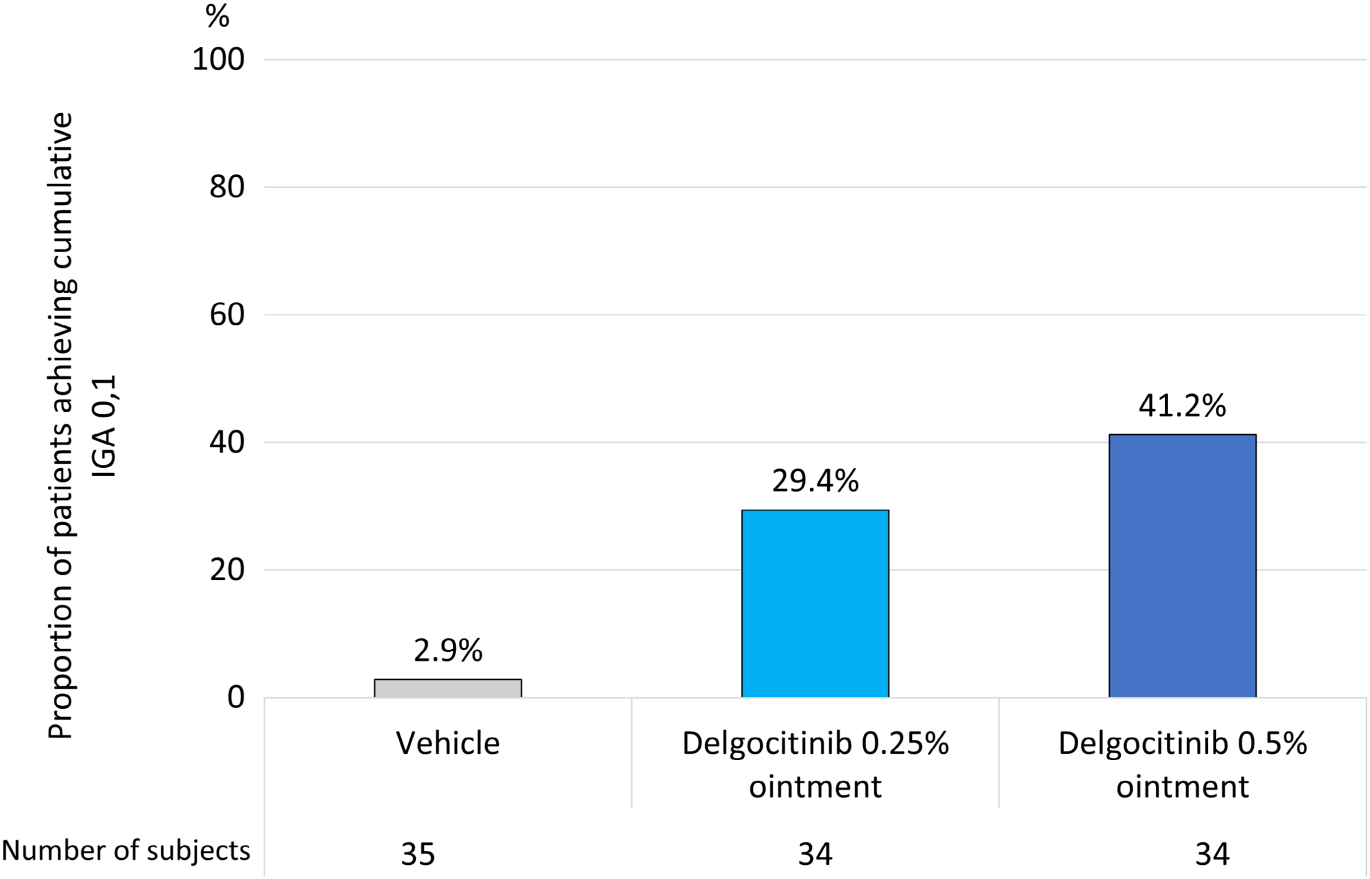
QBB2‐1, proportion of patients achieving cumulative IGA 0,1. IGA, investigator's global assessment.

**FIGURE 3:**
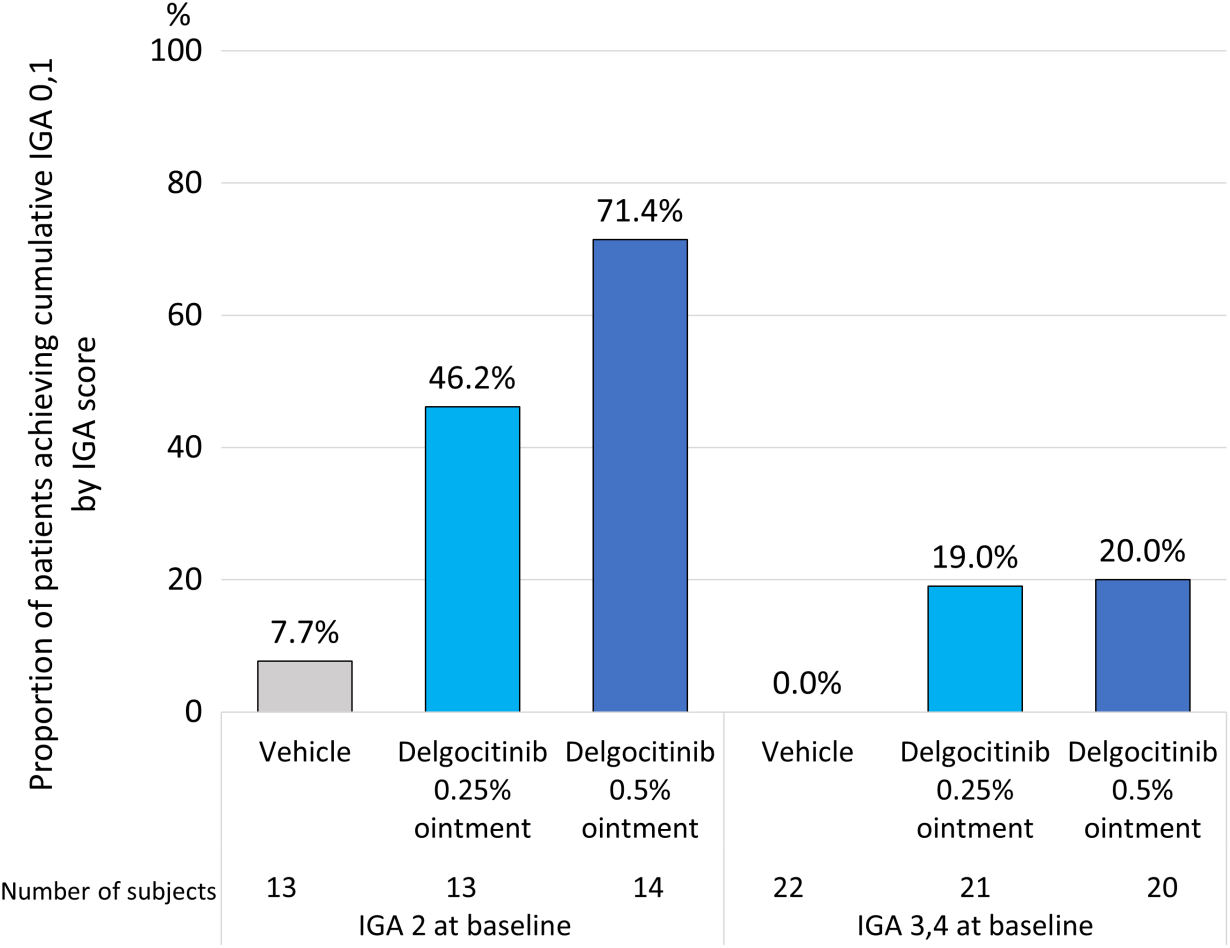
QBB2‐1, proportion of patients achieving cumulative IGA 0,1 by IGA score at baseline. IGA, investigator's global assessment.

The efficacy of delgocitinib 0.25% and 0.5% ointment, respectively, was assessed in the QBB2‐1 study population using cumulative IGA 0,1—i.e., the fraction of patients who achieved IGA 0 or 1 at least once during the treatment period. In the overall study population, the cumulative IGA 0,1 was 29.4% (10/34) in the 0.25% delgocitinib ointment treatment group and 41.2% (14/34) in the 0.5% delgocitinib ointment treatment group, and only 2.9% (1/35) in the vehicle treatment group (Figure 2). When analyzed by IGA score at baseline, the cumulative IGA 0,1 in the IGA 2 population subgroup was 46.2% (6/13) in the 0.25% delgocitinib ointment treatment group, 71.4% (10/14) in the 0.5% delgocitinib ointment treatment group, and 7.7% (1/13) in the vehicle treatment group (Figure 3). In contrast, only a minor difference was seen between the active treatment groups in the IGA 3,4 population subgroup. Here, the cumulative IGA 0,1 was 19.0% (4/21) in the 0.25% delgocitinib treatment group, 20.0% (4/20) in the 0.5% delgocitinib treatment group, and 0.0% (0/22) in the vehicle treatment group (Figure 3).

### Safety assessments

In the QBB2‐1 study, patients were randomized to receive either 0.25% delgocitinib ointment, 0.5% delgocitinib ointment, or vehicle throughout the trial, which allowed for analysis of a possible correlation between the frequency of TRAEs and treatment (Table 3). There were no differences between the three treatment groups regarding the number of patients that experienced TRAEs or the number of TRAE events that occurred during the study (Table 3). No difference in TRAEs between patients with mild (IGA 2) or moderate–severe (IGA 3,4) disease at baseline was seen (Table 3). This indicates that both delgocitinib ointment doses were well tolerated in this study population regardless of disease severity at the start of treatment.

**TABLE 3:** QBB2‐1, number of subjects with treatment‐related adverse events (TRAEs) and number of TRAEs.

TRAEs	IGA 2 (mild)						IGA 3,4 (moderate and severe)						Total					
	Vehicle		Delgocitinib 0.25% ointment		Delgocitinib 0.5% ointment		Vehicle		Delgocitinib 0.25% ointment		Delgocitinib 0.5% ointment		Vehicle		Delgocitinib 0.25% ointment		Delgocitinib 0.5% ointment	
*n*	13		13		14		22		21		20		35		34		34	
*N* (%)	e	*N*	e	*N*	e	*N*	e	*N*	e	*N*	e	*N*	e	*N*	e	*N*	e	*N*
Total	1	1 (7.7)	1	1 (7.7)	0	0 (0.0)	1	1 (4.5)	0	0 (0.0)	1	1 (5.0)	2	2 (5.7)	1	1 (2.9)	1	1 (2.9)
Infections and infestations	1	1 (7.7)	1	1 (7.7)	0	0 (0.0)	0	0 (0.0)	0	0 (0.0)	1	1 (5.0)	1	1 (2.9)	1	1 (2.9)	1	1 (2.9)
Impetigo	1	1 (7.7)	1	1 (7.7)	0	0 (0.0)	0	0 (0.0)	0	0 (0.0)	1	1 (5.0)	1	1 (2.9)	1	1 (2.9)	1	1 (2.9)
Skin and subcutaneous tissue disorders	0	0 (0.0)	0	0 (0.0)	0	0 (0.0)	1	1 (4.5)	0	0 (0.0)	0	0 (0.0)	1	1 (2.9)	0	0 (0.0)	0	0 (0.0)
Dermatitis contact	0	0 (0.0)	0	0 (0.0)	0	0 (0.0)	1	1 (4.5)	0	0 (0.0)	0	0 (0.0)	1	1 (2.9)	0	0 (0.0)	0	0 (0.0)

*Note*: TRAEs coded according to MedDRA/J 19.1.

Abbreviations: IGA, investigator’s global assessment; e, number of events; N, number of patients.

In the QBB4‐1 study, use of the 0.25% and 0.5% delgocitinib ointment doses in Part 2 of the study was not restricted to defined treatment groups as the two doses were used interchangeably depending on the patient's disease condition.^8^ In the QBB4‐1 study, the occurrence of TRAEs were therefore analyzed in two subgroups defined according to the dose or doses that had been applied during the study (0.25% and 0.5% delgocitinib ointment, respectively) (Supporting Information, Table S1 and Nakagawa et al.)^8^ Of the total study population of 134 patients, 109 received the 0.25% delgocitinib ointment dose at least once during the study, and 108 patients received the 0.5% delgocitinib ointment dose at least once during the study (Supporting Information, Table S1, and Nakagawa et al.^8^). A total of 13 TRAEs occurred in the QBB‐4‐1 study, the most common being application site folliculitis (Supporting Information, Table S1, and Nakagawa et al.^8^). 6 (5.5%) and 8 (7.4%) TRAEs were observed in the 0.25% and 0.5% delgocitinib ointment subgroups, respectively (Supporting Information, Table S1, and Nakagawa et al.^8^). The number of TRAEs in the two subgroups was assigned according the delgocitinib ointment dose that was used at the time of occurrence of the TRAE. In one instance, both the 0.25% and the 0.5% delgocitinib ointment treatment doses were applied to the same patient at the time of occurrence of a TRAE and this patient was consequently assigned to both treatment groups. No difference in the frequency or kind of TRAEs between the two subgroups was observed (Supporting Information, Table S1, and Nakagawa et al.).^8^

For the QBB4‐1 study population, two subgroups were defined according to rescue medication use (Table 4). 72 patients (54%) received rescue medication in addition to delgocitinib ointment treatment during Part 2 of the trial. Sixty‐two patients (46%) received only delgocitinib ointment treatment (Table 4). The frequency of TRAEs was similar in the two subgroups (*n* = 5 (8.1%) in the subgroup without rescue medication, *n* = 8 (11.1%) in the subgroup with rescue medication) (Table 4), indicating that the use of tacrolimus ointment and/or TCS as rescue medication in connection with delgocitinib ointment treatment was well tolerated in this pediatric study population.

**TABLE 4:** QBB4‐1, number of subjects with treatment‐related adverse events (TRAEs) and number of TRAEs by rescue medication.

	Without rescue medication	With rescue medication	Total
*n* (%)	62 (46.3)	72 (53.7)	134 (100.0)
TRAEs number (*n* [%])	5 (8.1)	8 (11.1)	13 (9.7)
General disorders and administration site conditions	1 (1.6)	3 (4.2)	4 (3.0)
Application site acne	1 (1.6)	1 (1.4)	2 (1.5)
Application site irritation	0 (0.0)	1 (1.4)	1 (0.7)
Application site urticaria	0 (0.0)	1 (1.4)	1 (0.7)
Infections and infestations	4 (6.5)	5 (6.9)	9 (6.7)
Application site folliculitis	1 (1.6)	3 (4.2)	4 (3.0)
Folliculitis	0 (0.0)	1 (1.4)	1 (0.7)
Herpes simplex	0 (0.0)	1 (1.4)	1 (0.7)
Impetigo	1 (1.6)	0 (0.0)	1 (0.7)
Molluscum contagiosum	2 (3.2)	0 (0.0)	2 (1.5)
Oral herpes	0 (0.0)	1 (1.4)	1 (0.7)
Skin infection	0 (0.0)	1 (1.4)	1 (0.7)
Neoplasms benign, malignant, and unspecified (including cysts and polyps)	0 (0.0)	1 (1.4)	1 (0.7)
Skin papilloma	0 (0.0)	1 (1.4)	1 (0.7)

*Note*: TRAEs were coded according to MedDRA/J V.21.0.

## DISCUSSION

Delgocitinib is a novel topical anti‐inflammatory drug, which ameliorates the three hallmark symptoms of AD—skin inflammation, pruritus, and reduced skin barrier function.^7^, ^8^, ^9^, ^10^, ^11^, ^12^ Two concentrations (0.25% and 0.5%) of delgocitinib ointment are available for pediatric AD in Japan.^5^ Based on available clinical data at the time of approval, the recommendation that the 0.25% formulation should be the normal dose used for children but also that the 0.5% dose could be used depending on the symptom severity was included in the delgocitinib package insert.^5^ The recommendation of using the 0.25% dose as the normal pediatric dose was based on the findings in the QBB2‐1 trial^7^ where no additional effect of the 0.5% dose compared to the 0.25% dose was seen on the primary efficacy endpoint (mEASI score percentage change from baseline at the end of the trial). However, a dose–response effect was seen on a secondary efficacy endpoint (percentage of subjects who achieved an IGA score of 0 or 1 at the end of the trial), which justified the recommendation to use the 0.5% dose when symptoms are severe, and the effect of the 0.25% dose is inadequate.

The symptoms of AD are known to fluctuate over time so the *post‐hoc* efficacy analyses shown here examine the effects of delgocitinib 0.25% and 0.5% ointment in the QBB2‐1 trial populations on the cumulative IGA 0,1 instead of IGA 0,1 at end‐of‐treatment as previously reported.^7^ The cumulative IGA 0,1 included all subjects who, at any time during the treatment period, achieved an IGA of 0 or 1. In the overall trial population, a similar dose–response effect as reported for end‐of‐treatment^7^ as was seen for cumulative IGA 0,1, with a higher proportion of subjects with cumulative IGA 0,1 the 0.5% treatment group compared to the 0.25% treatment group. This finding indicates that although cumulative IGA 0,1 may reflect the fluctuating nature of AD better than IGA 0,1 at end‐of treatment, it may be more relevant for trials with longer duration of treatment.

In contrast with the overall trial population, the analysis where cumulative IGA 0,1 was examined in two subgroups of the QBB2‐1 trial population with different levels of disease severity at baseline (IGA 2 and IGA 3,4, respectively) showed different dose–responses in the two otherwise well balanced subgroups. In the subgroup of patients with IGA 2 at baseline, a lower cumulative IGA 0,1 was achieved with the 0.25% delgocitinib ointment dose than the 0.5% dose (46.2% vs. 71.4%). In the subgroup of patients with IGA 3,4 at baseline, only a minor difference between the 0.25% and 0.5% doses was seen (19.0% vs. 20.0%). This indicates that in this study, the 0.5% delgocitinib ointment dose was more effective than the 0.25% dose for children with relatively mild AD symptoms at baseline but that the effect of the two doses were similar in patients with more severe disease. An additional study is needed to confirm if this dose–response relationship persists during longer periods of treatment where more fluctuations in AD symptoms are expected to occur. The results of the subgroup analysis also indicate that in patients with more severe symptoms at baseline, a combination of delgocitinib ointment and other AD medications may be required to achieve higher and perhaps more adequate treatment effects. This could include initial treatment with topical steroids to reduce disease severity before switching to delgocitinib for more long‐term use.

No difference between 0.25% and 0.5% delgocitinib ointment was detected regarding the frequency and kind of TRAEs in the overall trial population in the QBB2‐1 trial and no difference in the occurrence of TRAEs between subjects with IGA 2 or IGA 3,4 at baseline was seen. However, as simultaneous use of multiple medications may be needed for satisfactory AD management, the tolerability of the combined medication use needs to be assessed. In Part 2 of trial QBB4‐1, topical corticosteroids and/or tacrolimus ointment were allowed as rescue medication in combination with delgocitinib ointment treatment at the discretion of the investigator. Consequently, two subgroups from trial QBB4‐1 could be defined, one with patients who received treatment only with delgocitinib and one with patients who received rescue medication concomitantly with delgocitinib ointment treatment. As stated in the guidelines, the combination of drugs for AD is the basic approach to calm the inflammation quickly, and these results suggest that delgocitinib ointment can be used in combination with other drugs without safety concerns.^6^

While the patient populations included for the analyses presented here comprised pediatric AD patients 2–15 years of age, a recent open‐label un‐controlled study (JapicCTI‐205412) investigated the safety and efficacy of 0.25% and 0.5% delgicitonib ointment in Japanese infants with AD aged 6 to <24 months.^12^ The overall conclusions of the infant study were that delgocitinib ointment (0.25% and 0.5%) was well tolerated and effective for up to 52 weeks when applied to Japanese infants (6 to <24 months) with AD.^12^ With the caveat that the infant study and the 2 pediatric studies used in the analyses presented here may not be directly comparable due to differences in trial design and conduct, all three trials support the use of delgocitinib 0.25% and 0.5% in young children.

A limitation of this analysis is that no data on the possible correlation between TRAEs and delgocitinib ointment dose were collected. Previously, the number of TRAEs in trial QBB4‐1 compared to the delgocitinib ointment used at the time of occurrence of a TRAE was reported but no clear correlation between delgocitinib ointment dose and TRAEs was seen.^8^

An overall limitation of this study is that it comprises *post‐hoc* analyses of data from short‐term treatment (4 weeks) of a relatively small number of patients. The compliance rate for study medications (delgocitinib and rescue medications) application may also be higher during clinical trials than in actual clinical practice. Further limitations include that efficacy was evaluated only as the cumulative IGA 0,1 achievement rate, and that safety and efficacy data for application of delgocitinib ointment and rescue medication to the same site were not collected in any of the trials.

In summary, further studies including a larger number of patients are needed to firmly establish the tolerability of delgocitinib ointment in children with AD, but the data indicate that delgocitinib 0.5% ointment may be a valuable and more effective option than the 0.25% delgocitinib ointment for rapid relief of inflammation in mild cases of pediatric AD. Delgocitinib ointment was furthermore shown to be well‐tolerated in pediatric trial populations with no difference in the safety profiles of the 0.25% and 0.5% doses regardless of disease severity. There were also no differences in the safety profiles of subjects who were treated with delgocitinib ointment alone or in combination with other AD medications such as topical corticosteroids and/or tacrolimus ointment.

## AUTHOR CONTRIBUTIONS

Tatsuki Fukuie and Kenji Kabashima performed the interpretation of the result. Hiroyuki Toyama, Mai Tanaka and Katsuyo Ohashi‐Doi designed the study and wrote the manuscript. All authors read and approved the final manuscript.

## INFORMED CONSENT

Written informed consent was obtained from the parents or guardians of the patients.

## CONFLICT OF INTEREST STATEMENT

Tatsuki Fukuie received lecture fees from Abbvie, KYORIN Pharmaceutical, Maruho, Novartis, Otsuka Pharmaceutical, Thermo Fisher Diagnostic, Torii Pharmaceutical, and Viatris. He participated on the advisory board of Torii Pharmaceutical. Kenji Kabashima received grants from Japan Tobacco Inc., Kyowa Kirin, LEO Pharma, Maruho, Mitsubishi Tanabe Pharma, Ono Pharmaceutical, Sun Pharma, Procter & Gamble, Taiho Pharma and Torii Pharmaceutical. Hiroyuki Toyama, Mai Tanaka, and Katsuyo Ohashi‐Doi are employees of Torii Pharmaceutical Co., Ltd.

## ETHICS STATEMENT

QBB2‐1 and QBB4‐1 were conducted in compliance with Good Clinical Practice and the Declaration of Helsinki and were approved by the investigational review committee of each medical institution.

## Supporting information


Table S1.
