# Supplementary material for: Post‐hoc safety/efficacy analyses from pediatric delgocitinib atopic dermatitis trials
Source: Pediatr Int. 2024 Oct 7;66(1):e15798. doi: 10.1111/ped.15798 (PMC11580369; doi:10.1111/ped.15798)
Supplement: Supplementary file 1 — Table S1. [file PED-66-e15798-s001.docx]

**Supplementary Material**

**Table S1:** QBB4-1, number of patients with treatment-related adverse

events (TRAEs) and number of TRAEs by delgocitinib ointment concentration (adapted from “Supplementary Table VII” in Nakagawa et al, 2021^7^). TRAEs were coded according to MedDRA/J V.21.0.

* Number of patients who used delgocitinib 0.25% or 0.5% ointment at least once during the treatment period
